# "Contagion" or "Infection"

**Published:** 1899-07-01

**Authors:** 


### The Hospital

A Journal of

The Medical Sciences and Hospital Administration.

Vol. XXVI.—No. 666.] Edited by Sir Henry Burdett, K.C.B., and Solomon C. Smith, M.D., M.R.C.P. [July 1, 1899.

### "Contagion" or "Infection."

In the late Albert Smith's almost forgotten "Physiology of a Medical Student," the hero is in due course described as attending the class of a "grinder," in order to be prepared for the final ordeal of his professional examinations. A prominent member of this class is "Mr. Meek." The grinder has been explaining the use of alkaline carbonates in irritant poisoning, and has told his pupils to say, if asked what they would do in such a case, assuming neither chalk nor whitening to be readily procurable, that they would scrape a whitewashed wall with a fire-shovel. "It sounds practical, and will please the examiners." Now, Mr. Meek, proceeds the grinder, "what would you do if you were called to a man who had hanged himself?" "Please, sir," replies Mr. Meek, "I would scrape the wall with a fire-shovel." Since this was written, Mr. Meek has been realised, over and over again, by amateur professors of sanitation, in whose muddled heads there is seldom any distinction, founded on the causes or modes of diffusion of different diseases, between the measures of prevention which should be adopted in the case of any of them. Many years ago, when physicians had first established that some forms of disease were liable to be communicated by the agency of polluted water, a great body of so-called engineers stepped forward, and told the doctors to stand out of the way. Nothing, they said, was wanted for the preservation of health but pure water; and the purification and protection of a town water supply were matters which must rest with engineers, and with which doctors could have no possible concern. So the engineers ceased to be engineers only, and became "sanitary" engineers, at least on the brass plates which adorned the doors of their offices, but beyond which their knowledge of "sanitation" could scarcely be said to penetrate. In like manner, when it was shown that disease might be produced by the inhalation of sewer gas, and that the presence of such gas might occasionally be revealed by a "bad smell," the amateur immediately set himself to work to discover this fairly obvious condition, even in the case of maladies with which it could have no probable connection. There is seldom a local outbreak of diphtheria, for example, in which some scandalised resident does not write to the non-medical press in order to explain that the cause is not far to seek, for that he himself, a few nights before the occurrence of the first case, had discovered a "pestilential odour" when walking within a mile of the residence of the patient. When it came to be known that contagious diseases are usually or always of microbial origin, and that microbes belong to the vegetable kingdom, manufacturing chemists began to bid for the confidence and the pence of the public, and, with their preparations of professedly bactericide power, to announce that they, and not the engineers, were the possessors of the veritable secret. In fact, Codlin was the friend, and not Short. They used the jargon of advertisers, "ful faire and fetysly," although the infinitely numerous varieties and conditions of microbial life, or the way in which these might be modified by external circumstances, were as utterly "unknowe" to them as the "Frensch of Parys" to Chaucer's Prioress. It is difficult to read the claims put forward by some of these people without some passing feeling of regret that the discipline, which our ancestors were wont to administer at a cart's tail, cannot even now be applied to some of the impudent pretenders who endeavour to fleece the public by promises of results which it is manifestly impossible to secure. We are not without hope that the career of the amateur or trading sanitarian may in some small degree be checked by the publication of a very instructive paper, which was lately read before the Royal Society by M. Haffkine, on the subject of "Preventive Inoculation." In this, he called attention to the difference between disease-producing bacteria which are truly parasitic, and soon perish when removed from their accustomed host, and those which are saprophytic, or capable, when so removed, of living and multiplying in almost any form of organic matter; and he showed that the precautions, such as isolation of the sick and destruction of the manifestly infective material immediately proceeding from them, which may be highly effective against the former, are practically useless against the latter, in which the patient, as a focus of infection, may be insignificant when compared with the cultivation which is going on around him, and from which, therefore, it is necessary to seek, either by inoculation or in some other way, a method of protection by which the sound may be rendered less liable to suffer from causes of disease which are too widely diffused to be destroyed in the sick-room, or to be excluded from the environment of the healthy. Even the engineer or the chemist, if he can but bring himself to understand the existence of this wide distinction between the causes of contagion and the causes of infection, will be less likely than at present to rush into the province of the doctor, and to flourish his specific in conditions in which it is not calculated to be useful.

